# Regular dental care in preschoolers in rural Southern Brazil

**DOI:** 10.11606/s1518-8787.2020054001686

**Published:** 2020-03-30

**Authors:** Adriana Vieira Camerini, Alexandre Emidio Ribeiro Silva, Silvio Omar Macedo Prietsch, Rodrigo Dalke Meucci, Mariane Pergher Soares, Vanusa Belarmino, Fabiana da Silva Fernandes

**Affiliations:** I Universidade Federal do Rio Grande Faculdade de Medicina Rio GrandeRS Brasil Universidade Federal do Rio Grande. Faculdade de Medicina. Rio Grande, RS, Brasil; II Universidade Federal de Pelotas Faculdade de Odontologia Departamento de Odontologia Social e Preventiva PelotasRS Brasil Universidade Federal de Pelotas. Faculdade de Odontologia. Departamento de Odontologia Social e Preventiva. Pelotas, RS, Brasil

**Keywords:** Child, Preschool, Dental Health Care, Health Education, Dental, Rural Health

## Abstract

**OBJECTIVE:**

To evaluate if factors related to the mother’s previous guidance on her children’s dental health and the school attendance of children influence the regular dental care of preschoolers living in the rural area of a municipality in Southern Brazil.

**METHODS:**

A population-based study was conducted with 264 children under five years of age and their mothers. Socioeconomic and behavioral data were collected using a questionnaire, and the children were subjected to dental health tests. The outcome was the regular use of dental services. The main exposure variables were children’s care in daycare centers or schools and maternal guidance on the child’s dental health. Poisson regression analysis with robust variance adjustment was used to estimate prevalence ratios, considering a 95% confidence interval.

**RESULTS:**

The prevalence of regular use was 11.4% (95%CI 7.5–15.2). In the adjusted analysis, the regular use of services was associated with the child attending day care center/school (PR = 2.44; 95%CI 1.38–4.34), and the mother received dental health guidance (PR = 4.13; 95%CI 1.77–9.61), even with control for socioeconomic, maternal and child variables.

**CONCLUSION:**

When mothers receive previous information on child dental health care and children attend schools or daycare centers, the likelihood of regular dental appointments in preschoolers living in rural locations increases.

## INTRODUCTION

The regular use of dental services by preschool children is essential for the practice of primary prevention measures in dental health^1^. Preventive dental appointments seem to decrease subsequent visits of young children related to dental problems and may make care more effective and less costly when compared with emergency or hospital care^2^. The American Association of Pediatric Dentistry (AAPD) recommends the first dental examination occur at the breakup of the first tooth and before 12 months of age, and this first appointment is essential for primary prevention and early intervention^1^.

The main disease that affects children in this age group is dental caries and, therefore, the risk assessment for it is the greatest concern. In this sense, each child may need appointments and regular clinical interventions, stipulated by the individual risk resulted of a professional assessment^1^. Moreover, other procedures, such as prophylaxis, fluoride applications, dental restorations, malocclusion treatment and preventive guidance, are performed during regular appointments.

Despite presenting unmet treatment needs, the search for dental care is low among preschoolers^3^. In Brazil, dental health policies have focused their actions on schoolchildren, whereas preschoolers are on the margins of specific policies, despite presenting a high percentage of untreated caries and marked socioeconomic inequality in the distribution of the disease^6^. Obstacles in the use of services, such as access or financial difficulties, non-perception of the need for treatment and fear of dental procedures, are commonly related to non-demand for dental care^7,8^. However, it has been shown that, even where access to services exists and where there is an effort to increase the supply of calls, demand is still below expectations, with inequalities in the use of services^9,10^.

Complex processes are related to the search for dental care, possibly related to individual and psychological factors and also with the social structure or health policies^11^. Moreover, behavioral aspects, both infant and maternal, may be related to the use of services by preschool children. The health care of the mother is very important in this age group, as well as mental resources and abilities; their perception of needs, previous experiences and health habits can influence both the dental health conditions of their children and the use of dental services^3,4,12^.

In this sense, the use of regular services has been significantly lower among younger children when compared with older children, which may be related to the lack of dental health education^12^. Scientific evidence has also shown that activities that include health education, access to fluoridated water and dietary guidance are modifiable risk factors for early caries reduction^15^. Although oral diseases are preventable, most mothers need to know more about the child’s dental visits and dental health care^16^.

In addition to the knowledge in dental health acquired during dental appointments, other situations can be facilitators in the educational process. Promotion of dental health in schools is one of the strategies to ensure that information is available to parents and children. Attending school or daycare can allow conditions related to the child’s dental health to be identified by professionals, allowing to reinforce, together with parents, the need to seek dental services and adopt healthier habits and behaviors, increasing the chance of regular visits to the dentist^17^.

Therefore, our study evaluated if factors related to the mother’s previous guidance on her children’s dental health and the school attendance of children influence the regular dental care of preschoolers living in the rural area of a municipality in Southern Brazil.

## METHOD

This was a cross-sectional study with preschoolers living in the rural area of the municipality of Rio Grande, RS, and part of a research consortium called “Health of the rural population from Rio Grande.” The municipality of Rio Grande is located in the Southern Half of Rio Grande do Sul, the poorest region of the state, distant 350 km from the capital, Porto Alegre. The rural area of the municipality consists of 24 census tracts. At the end of the field research, 4,189 households were identified in the rural area of the municipality of Rio Grande, 2,669 of which were permanently inhabited.

A pilot study was conducted previously to test the questionnaire. All rural census tracts were mapped, and routes were made to contemplate all households. In households with children under five years old, mothers and children were invited to participate.

The field research was conducted in two stages. In the first stage, all households were visited to identify the eligible population. At this time, between April and October 2017, questionnaires were applied for women of childbearing age and mothers of children under five years of age. The instruments were applied by trained interviewers using tablets and the RedCap Program (Research Electronic Data Capture)^18^. In the second stage, from August 2017 to May 2018, a researcher trained and calibrated for dental health according to the criteria proposed by the World Health Organization^19^ returned to the households and evaluated dental caries in children.

The outcome “regular use of dental services” was evaluated by an instrument already used in previous studies in Brazil^5,14,20^. Changes were made for the mother to respond to the child’s dental visits, tested in a pilot study. Thus, the study evaluated mainly the pattern of dental appointments of children based on maternal reporting.

The mother were oriented regarding the performance of the test: “I’m going to read a few sentences and I’d like you to tell which one best describes the child’s appointments with the dentist.” The mother could choose one of the following answers: “1. He/she never goes to a dentist”; “2. He/she goes to the dentist when he has pain or a problem in the teeth or gums”; “3. He/she goes to the dentist sometimes, having a problem or not”; “4. He goes to the dentist on a regular basis.” The outcome was dichotomized in non-regular use (responses 1 or 2) and regular use (responses 3 or 4).

The main exposure variables of our study were: 1. The mother has received prior guidance on the child’s dental health, evaluated by the question: “Have you received guidance on how to take care of the child’s teeth/mouth?” (“yes” or “no”) and complemented by the question: “Who gave this guidance?” (“doctor/pediatrician,” “dentist,” “health agent,” “family/relative” or “teacher”); 2. the frequency of children in day care centers or preschools, assessed by the question: “Does the child attend school, preschool or a daycare center?” (“yes” or “no”).

The covariates of our study were socioeconomic, maternal and child-related. Socioeconomic covariates were: family income in minimum wages (< 2, 2–3 or > 3) and if the family has health insurance (“yes” or “no”). Maternal ones were: age in years (≤ 30 or > 30), skin color (“white,” “black,” “brown” or “yellow”), schooling in complete years of study (0–4, 5–8 or ≥ 9), number of prenatal appointments (≤ 5 or ≥ 6), regular use of dental services (“yes” or “no”), dental appointment in pregnancy (“yes” or “no”) and type of service that you used in the last appointment (“public” or “private”). Maternal dental anxiety was evaluated by the Portuguese version of the Dental Anxiety Scale (DAS)^21^. She uses four questions with scores ranging between 1 and 5, whose sum ranges between 4 and 20. Indicators of moderate or extreme anxiety were considered the scores greater than or equal to 11. Finally, the covariates related to the child were: age in years (“< 2” or “2–5”), sex (“male” or “female”), frequency of brushing (“0–2” or “≥ 3” times), perception of dental health reported by the mother (“very bad,” “bad,” “regular/very good” or “good”), perception of the need for treatment of the child reported by the mother (“yes” or “no”), fear of dentist of the child as reported by the mother (“not afraid” or “afraid”). The variable related to the child’s dental health condition was the experience of caries measured by the ceo-d index.

The statistical program Stata 14.0 was used for the analyses performed in our study. Initially, a descriptive analysis of all the variables of our study was performed. The chi-square test with Yates correction was used for comparisons between proportions. Poisson regression analysis with robust variance adjustment was used to estimate prevalence ratios and 95% confidence intervals. The outcome was analyzed regarding the main exposure variables and adjusted for socioeconomic variables (block 1), maternal (block 2) and the child (block 3). The theoretical model that guided the analyses was Andersen’s behavioral model^22^, that considers the predisposing, capacity and necessity factors in the search for dental care. All variables with p value ≤ 0.20 after adjustment for variables of the same level and the previous level were maintained in the model. The statistical significance of each variable in the model was evaluated by the Wald test (p ≤ 0.05).

Our study was approved by the Ethics Committee in Applied Health Research of the Universidade Federal do Rio Grande (FURG) under opinion no. 44/2017. The mothers signed the informed consent form for themselves and their children before the interviews and exams.

## RESULTS

A total of 360 preschoolers were identified in the countryside. Of these, 343 participated in our study (4.3% of losses and refusals). A total of 264 children had information about the use of dental services. Of these, 229 (86.9%) had oral tests. Comparing the children who underwent and those who did not undergo the dental caries assessment test, there were no significant differences regarding the main variables of the study: family income, maternal education, maternal age, dental anxiety, sex, age, dental health perception and regular use of dental services by the child.

The prevalence of regular use of dental services was 11.4% (95%CI 7.5–15,2). Table 1 shows the initial characteristics of the groups. The mean age of the children was 2.1 years (SD = 1.3) and 16.5% attended school or daycare. The age of the participants ranged between 18 and 52 years, with an average of 28.6 years (SD = 6.6). The experience of early caries was 34.5% (95%CI 28.3–40.7) and the mean decayed teeth lost or filled (ceo-d) was 1.64 (SD = 3.0). The highest prevalence of regular use of dental services in preschoolers were among children from families with higher income (p = 0.037), with mothers with a lower degree of anxiety (p = 0.013), who regularly use dental services (p < 0.001), who consulted a dentist during the child’s pregnancy (p = 0.012), in children of older age (p = 0.008), who were not afraid of dentist (p = 0.024) and who brushed their teeth at least three times a day (p = 0.043).

**Table 1:** Analysis of proportions between variables of interest and regular use of services by children in rural area of Rio Grande, RS, 2018 (N = 264).

Variable			Use of service		

	N	%	Non-regular	Regular	p
Home					
Household income in minimum wages (n = 238)					0.037^a^
**<** 2	157	66.0	90.5	9.5	
2–3	57	24.0	87.7	12.3	
> 3	24	10.0	70.8	29.2	
Does family have any health insurance? (n = 258)					0.742
No	165	64.0	87.9	12.1	
Yes	93	36.0	89.3	10.7	

Maternal					
Age in years (n = 260)					0.167
≤ 30	160	61.5	90.6	9.4	
> 30	100	38.5	85.0	15.0	
Skin color (n = 260)					0.354
White	215	82.7	89.3	10.7	
Black, brown or yellow	45	17.3	84.4	15.6	
Educational in years (n = 260)					0.248
0–4	34	13.1	85.3	14.7	
5–8	115	44.2	92.2	7.8	
≥ 9	111	42.7	85.6	14.4	
Number of prenatal appointments (n = 252)					0.146^b^
≤ 5	36	14.3	97.2	2.8	
≥ 6	216	85.7	87.5	12.5	
Dental anxiety (n = 247)					0.013^b^
Little or slightly anxious	164	66.4	84.2	15.8	
Moderate or extremely anxious	83	33.6	95.2	4.8	
Regular use of dental services (n = 250)					< 0.001
No	153	61.2	94.1	5.9	
Yes	97	38.8	78.4	21.6	
Dental appointment during pregnancy (n = 257)					0.012
No	178	69.3	92.7	7.3	
Yes	79	30.7	82.3	17.7	
Type of service used in the last dental appointment (n = 235)					0.829
Heath center or mobile unit	121	51.5	86.8	13.2	
Private or health plan	114	48.5	87.7	12.3	

Child					
Sex (n = 264)					0.388
Male	134	50.8	90.3	9.7	
Female	130	49.2	86.9	13.1	
Age (n = 260)					0.008^b^
< 2 years	90	34.6	95.6	4.4	
2–5 years	170	65.4	84.7	15.3	
Child attends school or daycare? (n = 260)					< 0.001
No	217	83.5	92.6	7.4	
Yes	43	16.5	67.4	32.6	
Mother received guidance on child oral care? (n = 262)					< 0.001
No	164	62.6	96.3	3.7	
Yes	98	37.4	75.5	24.5	
Are you afraid of a dentist? (n = 154)					0.024
No	90	58.4	74.4	25.6	
Yes	64	41.6	89.1	10.9	
Child dental health perception (n = 262)					0.274^b^
Very good/good	225	85.9	87.6	12.4	
Regular/bad/very bad	37	14.1	95.6	5.4	
Perception of treatment need (n = 243)					0.653
No	168	69.1	88.7	11.3	
Yes	75	30.9	86.7	13.3	
Daily brushing frequency (n = 197)					0.043
Up to 2 times	112	56.9	90.2	9.8	
3 or more times	85	43.1	80.0	20.0	
Caries experience (n = 229)					0.435
ceo-d = 0	150	65.5	90.7	9.3	
ceo-d > 0	79	34.5	87.3	12.7	

^a^ linear trend chi-square test, statistical significance p ≤ 0.05

^b^ Fisher Test, statistical significance p ≤ 0.05

ceo-d: dental caries index in deciduous teeth

Table 2 shows the results of the crude and adjusted Poisson regression analysis between the outcome and the main exposure variables. The adjustment was made for socioeconomic, maternal and child variables. In the crude analysis of children attending day care or school (prevalence ratio [PR] = 4.41; 95%CI, 2.33–8.37) and the mother received guidance on the child’s dental health (PR = 6.69; 95%CI 2.83–15.83) were associated with the outcome. They maintained the association, even after control for the other blocks of variables, presenting PR = 2.44 (95%CI 1.38–4.34) for the frequency in daycare centers and preschools and PR = 4.13 (95%CI 1.77–9.61) for mothers oriented regarding child’s dental health.

**Table 2:** Adjusted Analysis of Poisson regression between variables of interest in relation to the regular use of dental services, Rio Grande, 2018 (N = 264).

Regular use of dental services												

Variables	Crude analysis			Block 1			Block 2			Block 3		
			
	PR	95%CI	p^a^	PR	95%CI	p^a^	PR	95%CI	p^a^	PR	95%CI	p^a^
Child attends school or daycare												
No	1			1			1			1		
Yes	4.41	2.33–8.37	< 0.001	3.24	1.70–6.15	< 0.001	3.32	1.76–6.27	< 0.001	2.44	1.38–4.34	0.002
Mother received guidance on child oral care												
No	1		< 0.001	1			1			1		
Yes	6.69	2.83–15.83		5.61	2.33–13.50	< 0.001	4.82	1.99–11.65	< 0.001	4.13	1.77–9.61	0.001

Note: variables of Block 1: adjusted between them and for socioeconomic variables (family income and maternal age); block 2: adjusted for variables in block 1 + maternal variables (regular use of dental services, pregnancy appointments, number of prenatal appointments, maternal dental anxiety); block 3: adjusted for blocks 1 and 2 and for the child’s variables (age, dentist fear, daily brushing frequency).

^a^ Wald Test, statistical significance ≤ 0.05.

PR: prevalence ratio; 95%CI: 95% confidence interval

## DISCUSSION

The prevalence of regular use of dental services by children under five years of age living in rural area of Rio Grande was 11.3%. Those who attend schools and day care centers and those whose mothers received guidance, mainly from physicians and dentists, regarding the dental health of children used dental services more regularly, regardless of maternal and individual factors.

Early appointments have been shown to be effective in reducing oral diseases, especially in high-risk individuals. Therefore, it is necessary to increase the proportion of children performing preventive dental visits. Health-promoting strategies have stimulated actions within communities and in the school environment. Moreover, including in these actions the caregivers of the child (their parents, health professionals or teachers) may be important in changing the behavior necessary to increase the proportion of children who use dental services^23^.

In our study, 16.5% of children attended day care centers and preschools. This percentage is low when compared with data from the continuous National Household Sample Survey (NHSS)^24^, which evaluated access to education in Brazil, revealing a frequency of 40% at the age of zero to three years and 88.9% in children aged four and five years in the Southern Region of the country. We observed that rural children benefited from access to schools and day care centers, resulting in greater regularity in dental appointments. Providing dental health education in schools is believed to help children develop personal skills, providing knowledge about dental health and promoting positive attitudes and healthy behaviors.

Some successful experiences outside Brazil involved health education integrating family members, training teachers and creating healthy environments at school. Moreover, the relationship of these actions with health services was also essential^17^. In Brazil, initiatives to promote the intersectoriality provided for in health actions within the Unified Health System have been developed through the School Health Program. Day care centers and rural public preschools are among the priority schools for the performance of the program^25^. A study by Piovesan et al.^12^ evaluated mothers’ participation in school activities and showed that not participating in them significantly increased the chance that the child had never consulted a dentist.

The formation of dental health habits occurs during childhood, which is the best age to acquire new behaviors and attitudes. A meta-analysis study showed that health education and health promotion interventions in children are effective in increasing dental visits, dental brushing behaviors and flossing after three months of intervention, having their maximum effect on children when compared with groups of adolescents and adults^26^. Dental health education programs for families showed that children who participated for 12 months were more likely to have preventive dental appointments and for restoration, as well as a lower chance of performing emergency appointments^27^.

In our study, children of mothers from rural areas who received previous dental health guidance were four times more likely to be regular users of dental services. Education has proved to be an effective strategy when included in health policies. Individuals with greater knowledge about health-disease processes are more apt for self-care and also for the transmission of knowledge to their children. Previous studies in urban areas found a strong relationship between preventive appointments and the receipt of dental health guidelines in children^5,13^. The orientation of the family regarding these care of the child can be performed by health professionals during appointments. School and other health professionals’ actions can also transmit knowledge and guidance, in addition to referring patients to services.

We observed an important role of the physician as director of this maternal counseling, and this professional is the first who usually contacts the patient who seeks the health service. We also observed that the health agent is underused by the community in the transmission of these knowledge, thus resulting in communities missing opportunities for preventive guidance on dental health problems and in the need to perform early appointments. Some studies have warned of the potential role of non-dentistry professionals in identifying children with dental health problems and forwarding health services for treatment^23^.

According to Harris et al.^11^, access to health knowledge can be acquired by formal or informal means and seems to be part of a social and community process. How the information will be processed by individuals will depend on the recipient’s educational level, among other individual capacities. Health services and policies are responsible for providing understandable and reliable information, reaching individuals so that they can properly apply it, thus reducing the factors that are involved in inequality in preventive use of services dental health.

A systematic review and meta-analysis study showed that rural residents used regular dental services less when compared with urban areas (odds ratio = 0.87; 95%CI 0.76–0.97)^10^. Moreover, children in the rural area may be more likely to miss preventive appointments when compared with those in the urban area^9^. In the municipality of Rio Grande, RS, the rural area has full coverage of the Family Health Strategy, characterized by providing primary health care to the population registered in health units. However, it does not have dental health teams in its basic health units, and the care was done by mobile units, sporadically. The presence of the dental health team working together with the family health teams has been pointed out in the literature as an important factor for greater use of dental health services by children up to three years when compared with those who consulted traditional health units^3^, showing the importance of organizing health services respecting the precepts of primary care.

In our study, approximately one third of the children had some experience of caries. The presence of dental caries is the main reason to seek dental service^14,28^. However, caries prevention depends on early identification of at-risk children and preventive care such as application of sealants and fluoride^29^. In our study, early caries was not associated with the regular use of dental services by children, confirming findings from previous studies in which children who regularly use these services did not have their routine or preventive appointments motivated by oral problems^5^. In this sense, the average of teeth affected by caries in children who consulted by check-up may be significantly lower than those who consult for specific reasons. Recent findings indicate that, as the number of dental appointments and educational actions for prevention increases, there is a decrease in the number of carious lesions in children, observed both in urban and rural environments^30^.

All studies have limitations and positive points. Two positive aspects of our study should be considered. The first is the population-based nature of our study, different from previous studies that evaluated children enrolled in schools or during vaccination campaigns. We could, therefore, evaluate the difference between children who were in school and those who out. Another positive point is the data collection. The interviewers were trained to apply the questionnaire and trained and calibrated to obtain dental health information, minimizing the possibility of information bias. Regarding limitations, the questions that evaluated the main exposures of our study may have been limited in gathering information on dental health education of mothers and how these guidelines affect other dental health behaviors. Another limitation of our study is that the information of the child was reported by the mother, and there is the possibility of the occurrence of recall bias.

We concluded that just over 11% of rural preschoolers used dental services regularly. Children attending day care or school and whose mothers received previous information on dental health care had a higher frequency of regular use of dental services. These results reinforce the need to structure health services and act intersectorally to ensure effective access to dental health through family health teams. In addition to instructing mothers about the importance of early care for children’s dental health, it is also necessary to prioritize children who do not go to school yet and depend essentially on maternal care.
