# Supplementary material for: Regular dental care in preschoolers in rural Southern Brazil
Source: Rev Saude Publica. 2020 Mar 30;54:37. doi: 10.11606/s1518-8787.2020054001686 (PMC7112741; doi:10.11606/s1518-8787.2020054001686)
Supplement: Translation

# Translation: Atendimento odontológico regular em pré-escolares da área rural do Sul do Brasil

**Authors:** Adriana Vieira Camerini, Alexandre Emidio Ribeiro Silva, Silvio Omar Macedo Prietsch, Rodrigo Dalke Meucci, Mariane Pergher Soares, Vanusa Belarmino, Fabiana da Silva Fernandes

**Affiliations:** I Brasil Universidade Federal do Rio Grande. Faculdade de Medicina. Rio Grande, RS, Brasil; II Brasil Universidade Federal de Pelotas. Faculdade de Odontologia. Departamento de Odontologia Social e Preventiva. Pelotas, RS, Brasil

**Keywords:** Pré-Escolar, Serviços de Saúde Bucal, Educação em Saúde Bucal, Saúde da População Rural

## Abstract

**OBJETIVO:**

Avaliar se fatores relacionados à orientação prévia da mãe sobre saúde bucal dos seus filhos e a frequência escolar das crianças influenciam o atendimento odontológico regular de pré-escolares moradores da área rural de um município do Sul do Brasil.

**MÉTODOS:**

Um estudo de base populacional foi conduzido com 264 crianças menores de cinco anos e suas mães. Dados socioeconômicos e comportamentais foram coletados por meio de questionário e as crianças foram submetidas a exames de saúde bucal. O desfecho foi o uso regular de serviços odontológicos. As variáveis de exposição principais foram o atendimento infantil em creches ou escolas e orientação materna sobre a saúde bucal da criança. A análise de regressão de Poisson com ajuste robusto de variância foi utilizada para estimar as razões de prevalência e intervalos de confiança de 95%.

**RESULTADOS:**

A prevalência de uso regular foi 11,4% (IC95% 7,5–15,2). Na análise ajustada o uso regular de serviços foi associado à criança frequentar creche/escola (RP = 2,44; IC95% 1,38–4,34) e a mãe ter recebido orientação de saúde bucal (RP = 4,13; IC95% 1,77–9,61), mesmo com controle para variáveis socioeconômicas, maternas e da criança.

**CONCLUSÃO:**

Quando as mães recebem informações prévias sobre os cuidados com a saúde bucal infantil e as crianças frequentam escolas ou creches, aumenta a probabilidade de consultas odontológicas regulares em pré-escolares residentes em localidades rurais.

## INTRODUÇÃO

A utilização regular de serviços odontológicos por crianças pré-escolares é de fundamental importância para a prática de medidas de prevenção primária em saúde bucal^1^. Consultas odontológicas preventivas em crianças pequenas parecem diminuir visitas subsequentes relacionadas a problemas odontológicos e podem tornar o atendimento mais efetivo e menos oneroso quando comparadas com atendimentos de emergência ou hospitalares^2^. A Associação Americana de Odontopediatria (AAPD) preconiza que o primeiro exame odontológico ocorra no rompimento do primeiro dente e antes dos 12 meses de idade, sendo essa primeira consulta essencial para a prevenção primária e intervenção precoce^1^.

A principal doença que afeta crianças dessa faixa etária é a cárie dentária e, portanto, a avaliação de risco para ela é a maior preocupação. Neste sentido, cada criança poderá ter uma necessidade de consultas e periodicidade das intervenções clínicas, estipulada pela avaliação profissional de risco individual^1^. Além disso, outros procedimentos, como profilaxias, aplicações de flúor, restaurações dentárias, tratamento de mal oclusões e orientações preventivas, são realizados durante as consultas regulares.

Apesar de apresentarem necessidades de tratamento não satisfeitas, a procura por atendimento odontológico é baixa entre pré-escolares^3^. No Brasil, as políticas de saúde bucal têm focado suas ações em escolares, enquanto os pré-escolares ficam à margem de políticas específicas, apesar de apresentar alto percentual de cáries não tratadas e marcante desigualdade socioeconômica na distribuição da doença^6^. Obstáculos na utilização dos serviços, como dificuldades de acesso ou financeiras, não percepção da necessidade do tratamento e medo dos procedimentos odontológicos, estão comumente relacionados à não procura por atendimento odontológico^7,8^. No entanto, tem se demonstrado que, mesmo onde o acesso aos serviços existe e onde há um esforço para aumentar a oferta de atendimentos, a procura ainda fica abaixo do esperado, persistindo desigualdades na utilização dos serviços^9,10^.

Processos complexos estão relacionados com a procura por atendimento odontológico, podendo estar relacionados a fatores individuais e psicológicos e também com a estrutura social ou políticas de saúde^11^. Além disso, aspectos comportamentais, tanto infantis quanto maternos, podem estar relacionados ao uso de serviços por crianças pré-escolares. Os cuidados de saúde da mãe são muito importantes nessa faixa etária, assim como os recursos e capacidades mentais; a sua percepção de necessidades, as experiências anteriores e os hábitos de saúde podem influenciar tanto as condições de saúde bucal de seus filhos como a utilização dos serviços odontológicos^3,4,12^.

Nesse sentido, a utilização de serviços regulares tem se mostrado significativamente mais baixa entre crianças mais jovens quando comparadas com as mais velhas, o que pode estar relacionado à falta de educação em saúde bucal^12^. Evidências científicas têm demonstrado também que atividades que incluam educação em saúde, acesso a água fluoretada e orientação dietética são fatores de risco modificáveis para redução de cárie precoce^15^. Apesar de as doenças bucais serem passíveis de prevenção, grande parte das mães necessita conhecer mais sobre as visitas odontológicas e cuidados com a saúde bucal da criança^16^.

Além do conhecimento em saúde bucal adquirido durante as consultas odontológicas, outras situações podem ser facilitadoras no processo educativo. Uma das estratégias para garantir que as informações estejam disponíveis para os pais e crianças é a promoção de saúde bucal nas escolas. Frequentar a escola ou creche pode permitir que condições relacionadas à saúde bucal da criança sejam identificadas pelos profissionais, permitindo reforçar, junto com os pais, a necessidade de procurar os serviços de odontologia e adotar hábitos e comportamentos mais saudáveis, aumentando a chance de visitas regulares ao dentista^17^.

Portanto, este estudo teve como objetivos avaliar se fatores relacionados à orientação prévia de mães sobre saúde bucal dos seus filhos e se a criança frequentar escola ou creche influenciam o atendimento odontológico regular de pré-escolares moradores de comunidades rurais de um município do Sul do Brasil.

## MÉTODO

Este foi um estudo transversal de caráter censitário com pré-escolares residentes na área rural do município de Rio Grande, RS, e fez parte de um consórcio de pesquisa chamado “Saúde da população rural Riograndina”. O município de Rio Grande localiza-se na Metade Sul do Rio Grande do Sul, a região mais pobre do estado, distante 350 km da capital, Porto Alegre. A zona rural do município é constituída por 24 setores censitários. Ao final do trabalho de campo, foram identificados 4.189 domicílios na área rural do município de Rio Grande, sendo 2.669 domicílios permanentemente habitados.

Um estudo-piloto foi realizado previamente para testar o questionário. Foi feito um mapeamento dos setores censitários rurais e realizados trajetos para contemplar todos os domicílios. Nos domicílios onde haviam crianças menores de cinco anos, as mães e as crianças foram convidadas a participar.

O trabalho de campo foi feito em 2 etapas. Na primeira etapa da pesquisa, todos os domicílios foram visitados para identificação da população elegível. Nessa ocasião, entre abril e outubro de 2017, foram aplicados questionários para mulheres em idade fértil e mães de crianças menores de cinco anos. Os instrumentos foram aplicados por entrevistadoras treinadas, utilizando *tablets* e o programa *RedCap* (*Research Electronic Data Capture*)^18^. Na segunda etapa, de agosto de 2017 a maio de 2018, uma pesquisadora, treinada e calibrada para a saúde bucal conforme os critérios propostos pela Organização Mundial de Saúde^19^, voltou nos domicílios e avaliou cárie dentária nas crianças.

O desfecho “utilização regular de serviços odontológicos” foi avaliado por um instrumento já empregado em estudos anteriores no Brasil^5,14,20^. Foram feitas adaptações para a mãe responder sobre as visitas odontológicas da criança, testadas em estudo-piloto. Dessa maneira, o estudo pretendeu avaliar principalmente o padrão das consultas odontológicas das crianças a partir do relato materno.

Foi realizada a pergunta: “Eu vou ler algumas frases e gostaria que a senhora dissesse qual delas descreve melhor as consultas da criança com o dentista”. A mãe poderia optar por uma das seguintes respostas: “1. Ele (a) nunca vai ao dentista”; “2. Ele (a) vai ao dentista quando tem dor ou um problema nos dentes ou na gengiva”; “3. Ele (a) vai ao dentista às vezes, tendo um problema ou não”; “4. Ele(a) vai ao dentista de forma regular”. O desfecho foi dicotomizado em uso não regular (respostas 1 ou 2) e uso regular (respostas 3 ou 4).

As principais variáveis de exposição do estudo foram: 1. a mãe ter recebido orientação prévia sobre saúde bucal da criança, avaliada pela pergunta: “A senhora já recebeu orientação sobre como cuidar dos dentes/boca da criança?” (sim ou não) e complementada pela pergunta: “Quem deu essa orientação?” (médico/ pediatra, dentista, agente de saúde, familiar/parente ou professora); 2. a frequência da criança em creches ou pré-escolas, avaliada pela pergunta: “A criança frequenta escola, escolinha ou creche?” (sim ou não).

As covariáveis do estudo foram socioeconômicas, maternas e relacionadas à criança. As socioeconômicas foram: renda familiar em salários mínimos (< 2, 2–3 ou > 3) e se a família possui plano de saúde (sim ou não). Já as maternas foram: idade em anos (≤ 30 ou > 30), cor da pele (branca, preta, parda ou amarela), escolaridade em anos completos de estudo (0–4, 5–8 ou ≥ 9), número de consultas pré-natal (≤ 5 ou ≥ 6), uso regular de serviços odontológicos (sim ou não), consulta odontológica na gravidez (sim ou não) e tipo de serviço que utilizou na última consulta (público ou privado). A ansiedade dental materna foi avaliada pela versão em português da escala *Dental Anxiety Scale* (DAS)^21^. Ela utiliza quatro perguntas com pontuações que vão de 1 a 5, cujo somatório varia de 4 a 20. Foram consideradas indicadoras de ansiedade moderada ou extrema as pontuações maiores ou iguais a 11. Por fim, as covariáveis relacionadas à criança foram: idade em anos (< 2 ou 2–5), sexo (masculino ou feminino), frequência de escovação (0–2 ou ≥ 3 vezes), percepção da saúde bucal relatada pela mãe (muito ruim, ruim, regular/muito boa ou boa), percepção de necessidade de tratamento da criança relatada pela mãe (sim ou não), medo de dentista da criança conforme relatado pela mãe (não tem medo ou tem medo). A variável relacionada com a condição de saúde bucal da criança foi a experiência de cárie medida pelo índice ceo-d.

Para as análises realizadas no estudo, foi utilizado o programa estatístico Stata 14.0. Inicialmente, foi feita uma análise descritiva de todas as variáveis do estudo. Para as comparações entre as proporções, foi utilizado o teste do qui-quadrado com correção de Yates. A análise de regressão de Poisson com ajuste robusto da variância foi utilizada para estimar as razões de prevalência e intervalos de confiança de 95% (IC95%). O desfecho foi analisado em relação às variáveis de exposição principais e ajustado para variáveis socioeconômicas (bloco 1), maternas (bloco 2) e da criança (bloco 3). O modelo teórico que guiou as análises foi o modelo comportamental de Andersen^22^, que considera os fatores predisponentes, capacitantes e de necessidade na procura por atendimento odontológico. Foram mantidas no modelo todas as variáveis com valor de p ≤ 0,20 após ajuste para variáveis do mesmo nível e do nível anterior. A significância estatística de cada variável no modelo foi avaliada pelo teste de Wald (p ≤ 0,05).

O presente estudo foi aprovado pelo Comitê de Ética do Conselho de Ética em Pesquisas Aplicadas à Saúde (CEPAS) da Universidade Federal do Rio Grande (FURG) sob o parecer número 44/2017. As mães assinaram o termo de consentimento para si e seus filhos antes da realização das entrevistas e dos exames.

## RESULTADOS

Foram identificados 360 pré-escolares na zona rural. Desses, 343 participaram do estudo (4,3% de perdas e recusas). Um total de 264 crianças tinha informação sobre o uso de serviços odontológicos. Dessas, 229 (86,9%) fizeram exames bucais. Comparando-se as crianças que realizaram e as que não realizaram o exame para avaliação de cárie dentária, não houve diferenças significativas em relação às principais variáveis do estudo: renda familiar, escolaridade materna, idade materna, ansiedade dental materna, sexo, idade, percepção de saúde bucal e uso regular de serviços odontológicos pela criança.

A prevalência de utilização regular de serviços odontológicos foi de 11,4% (IC95% 7,5–15,2). As características das crianças e mães estão apresentadas na Tabela 1. A média de idade das crianças foi de 2,1 anos (DP = 1,3) e 16,5% frequentavam escola ou creche. A idade materna variou entre 18 e 52 anos, com a média de 28,6 anos (DP = 6,6 anos). A experiência de cárie precoce foi de 34,5% (IC95% 28,3–40,7) e a média de dentes cariados perdidos ou obturados (ceo-d) foi de 1,64 (DP = 3,0). As maiores prevalências de uso regular de serviços odontológicos em pré-escolares foram entre crianças de famílias com maior renda (p = 0,037), com mães com menor grau de ansiedade (p = 0,013), que usam regularmente serviços odontológicos (p < 0,001), que consultaram um dentista durante a gravidez da criança (p = 0,012), em crianças de maior idade (p = 0,008), que não tinham medo de dentista (p = 0,024) e que escovavam os dentes no mínimo três vezes ao dia (p = 0,043).

**Tabela 1:** Análise de proporções entre variáveis de interesse e uso regular de serviços por crianças da zona rural de Rio Grande, RS, 2018 (N = 264).

Variável			Uso de serviços		

	N	%	Não regular	Regular	p
Domiciliar					
Renda familiar em salários mínimos (n = 238)					0,037^a^
< 2	157	66,0	90,5	9,5	
2–3	57	24,0	87,7	12,3	
> 3	24	10,0	70,8	29,2	
Família tem plano de saúde? (n = 258)					0,742
Não	165	64,0	87,9	12,1	
Sim	93	36,0	89,3	10,7	

Materna					
Idade em anos (n = 260)					0,167
≤ 30	160	61,5	90,6	9,4	
> 30	100	38,5	85,0	15,0	
Cor da pele (n = 260)					0,354
Branca	215	82,7	89,3	10,7	
Preta, parda ou amarela	45	17,3	84,4	15,6	
Escolaridade em anos (n = 260)					0,248
0–4	34	13,1	85,3	14,7	
5–8	115	44,2	92,2	7,8	
≥ 9	111	42,7	85,6	14,4	
Número de consultas pré-natal (n = 252)					0,146^b^
≤ 5	36	14,3	97,2	2,8	
≥ 6	216	85,7	87,5	12,5	
Ansiedade dental (n = 247)					0,013^b^
Pouco ou levemente ansiosa	164	66,4	84,2	15,8	
Moderada ou extremamente ansiosa	83	33,6	95,2	4,8	
Uso regular de serviços odontológicos (n = 250)					< 0,001
Não	153	61,2	94,1	5,9	
Sim	97	38,8	78,4	21,6	
Consulta odontológica durante a gravidez (n = 257)					0,012
Não	178	69,3	92,7	7,3	
Sim	79	30,7	82,3	17,7	
Tipo de serviço utilizado na última consulta odontológica (n = 235)					0,829
Posto ou unidade móvel	121	51,5	86,8	13,2	
Particular ou convênio	114	48,5	87,7	12,3	

Criança					
Sexo (n = 264)					0,388
Masculino	134	50,8	90,3	9,7	
Feminino	130	49,2	86,9	13,1	
Idade (n = 260)					0,008^b^
< 2 anos	90	34,6	95,6	4,4	
2–5 anos	170	65,4	84,7	15,3	
Frequenta escola ou creche? (n = 260)					< 0,001
Não	217	83,5	92,6	7,4	
Sim	43	16,5	67,4	32,6	
Mãe recebeu orientação sobre cuidados com a boca da criança? (n = 262)					< 0,001
Não	164	62,6	96,3	3,7	
Sim	98	37,4	75,5	24,5	
Tem medo de dentista? (n = 154)					0,024
Não	90	58,4	74,4	25,6	
Sim	64	41,6	89,1	10,9	
Percepção de saúde bucal da criança (n = 262)					0,274^b^
Muito boa/boa	225	85,9	87,6	12,4	
Regular/ruim/muito ruim	37	14,1	95,6	5,4	
Percepção de necessidade de tratamento (n = 243)					0,653
Não	168	69,1	88,7	11,3	
Sim	75	30,9	86,7	13,3	
Frequência de escovação diária (n = 197)					0,043
Até 2 vezes	112	56,9	90,2	9,8	
3 ou mais vezes	85	43,1	80,0	20,0	
Experiência de cárie (n = 229)					0,435
ceo-d = 0	150	65,5	90,7	9,3	
ceo-d > 0	79	34,5	87,3	12,7	

^a^ Teste do qui-quadrado de tendência linear, significância estatística p ≤ 0,05

^b^ Teste de Fisher, significância estatística p ≤ 0,05

ceo-d: índice de cárie dentária em dentes decíduos

A Tabela 2 mostra os resultados da análise bruta e ajustada de regressão de Poisson entre o desfecho e as variáveis de exposição principais. O ajuste foi feito para variáveis socioeconômicas, maternas e da criança. Na análise bruta a crianças frequentar creche ou escola (razão de prevalências [RP] = 4,41; IC95% 2,33–8,37) e a mãe ter recebido orientações sobre a saúde bucal da criança (RP = 6,69; IC95% 2,83–15,83) estiveram associadas ao desfecho. Elas mantiveram a associação, mesmo após controle para os demais blocos de variáveis, apresentando RP = 2,44 (IC95% 1,38–4,34) para a frequência em creches e pré-escolas e RP = 4,13 (IC95% 1,77–9,61) para mães orientadas em relação à saúde bucal da criança.

**Tabela 2:** Análise ajustada de regressão de Poisson entre variáveis de interesse em relação ao uso regular de serviços odontológicos, Rio Grande, 2018 (N = 264).

Uso regular de serviços odontológicos												

Variáveis	Análise bruta			Bloco 1			Bloco 2			Bloco 3		
			
	RP	IC95%	p^a^	RP	IC95%	p^a^	RP	IC95%	p^a^	RP	IC95%	p^a^
Criança frequenta escola ou creche												
Não	1			1			1			1		
Sim	4,41	2,33–8,37	< 0,001	3,24	1,70–6,15	< 0,001	3,32	1,76–6,27	< 0,001	2,44	1,38–4,34	0,002
Mãe recebeu orientações sobre cuidados com a boca da criança												
Não	1		< 0,001	1			1			1		
Sim	6,69	2,83–15,83		5,61	2,33–13,50	< 0,001	4,82	1,99–11,65	< 0,001	4,13	1,77–9,61	0,001

Nota: variáveis do Bloco 1: ajustadas entre elas e para variáveis socioeconômicas (renda familiar e idade materna); bloco 2: ajustadas para variáveis do bloco 1 + variáveis maternas (uso regular de serviços odontológicos, consultas na gravidez, número de consultas pré-natal, ansiedade dental materna); bloco3: ajustadas para blocos 1 e 2 e para as variáveis da criança (idade, medo de dentista, frequência de escovação diária).

^a^ Teste de Wald, significância estatística ≤ 0,05.

RP: razão de prevalências; IC95%: intervalo de confiança de 95%

## DISCUSSÃO

A prevalência de uso regular de serviços odontológicos por crianças menores de cinco anos que moram na zona rural de Rio Grande foi de 11,3%. As que frequentam escolas e creches e aquelas cujas mães receberam orientações, principalmente de médicos e dentistas, em relação à saúde bucal dos filhos utilizaram com maior regularidade os serviços odontológicos, independentemente de fatores socioeconômicos, maternos e individuais.

A realização de consultas precoces tem se mostrado eficaz na redução de doenças bucais, principalmente em indivíduos de alto risco. Portanto, é necessário aumentar a proporção de crianças realizando visitas odontológicas preventivas. Estratégias promotoras de saúde têm estimulado ações dentro das comunidades e no ambiente escolar. Além disso, incluir nessas ações os cuidadores da criança (sejam eles pais, profissionais de saúde ou professores) pode ser importante na mudança de comportamento necessária para aumentar a proporção de crianças que usam serviços odontológicos^23^.

Neste estudo, 16,5% das crianças frequentavam creches e pré-escolas. Esse percentual é baixo quando comparado com dados da Pesquisa Nacional por Amostra de Domicílios (PNAD) contínua^24^, que avaliou o acesso à educação no Brasil, revelando uma frequência de 40% na idade de zero a três anos e de 88,9% em crianças de quatro e cinco anos na Região Sul do país. Foi observado que as crianças rurais se beneficiaram com o acesso a escolas e creches, resultando em maior regularidade nas consultas odontológicas. Acredita-se que proporcionar educação sobre saúde bucal nas escolas ajude as crianças a desenvolver habilidades pessoais, fornecendo conhecimento sobre a saúde bucal e promovendo atitudes positivas e comportamentos saudáveis.

Algumas experiências bem-sucedidas fora do Brasil envolveram educação em saúde integrando familiares, treinando professores e criando ambientes saudáveis na escola. Além disso, a relação dessas ações com os serviços de saúde também foram imprescindíveis^17^. No Brasil, iniciativas para promover a intersetorialidade prevista nas ações de saúde dentro do Sistema Único de Saúde têm sido desenvolvidas por meio do Programa Saúde na Escola. Entre as escolas prioritárias para a atuação do programa, estão as creches e pré-escolas públicas rurais^25^. Um estudo de Piovesan et al.^12^ avaliou a participação das mães em atividades escolares e mostrou que não participar delas aumentava significativamente a chance de o filho nunca ter consultado um dentista.

A formação de hábitos de saúde bucal acontece durante a infância, sendo essa a melhor idade para adquirir novos comportamentos e atitudes. Um estudo de metanálise mostrou que a educação em saúde e intervenções de promoção de saúde em crianças são efetivas no aumento de visitas odontológicas, comportamentos de escovação dentária e uso de fio dental após três meses da intervenção, tendo seu máximo efeito nas crianças, quando comparadas a grupos de adolescentes e adultos^26^. Programas de educação em saúde bucal para famílias mostraram que as crianças que participaram durante 12 meses tinham maior chance de consultas odontológicas preventivas e para restauração, assim como uma menor chance de realizar consultas de emergência^27^.

No presente estudo, as mães de zonas rurais que receberam orientação prévia de saúde bucal tinham uma probabilidade quatro vezes maior de os filhos serem usuários regulares dos serviços odontológicos. A educação tem se mostrado uma estratégia efetiva quando incluída nas políticas de saúde. Indivíduos com maior conhecimento sobre os processos saúde-doença estão mais aptos ao autocuidado e também à transmissão de conhecimentos aos filhos. Estudos anteriores em zonas urbanas encontraram forte relação entre consultas preventivas e o recebimento de orientações de saúde bucal em crianças^5,13^. A orientação da família em relação a esses cuidados da criança pode ser realizada por profissionais de saúde durante as consultas. Ações escolares e de outros profissionais de saúde também podem transmitir conhecimentos e orientações, além de encaminhar os pacientes aos serviços.

Foi observado neste estudo um importante papel do médico como realizador deste aconselhamento materno, sendo esse profissional o primeiro que normalmente faz contato com o paciente que procura o serviço de saúde. Também é possível perceber uma subutilização do agente comunitário de saúde na transmissão desses conhecimentos, perdendo oportunidades de orientação preventiva sobre problemas de saúde bucal e sobre a necessidade de realizar consultas precoces. Alguns estudos têm alertado para o papel potencial de profissionais que não são da odontologia em identificar as crianças com problemas de saúde bucal e encaminhar aos serviços de saúde para tratamento^23^.

Segundo Harris et al.^11^, o acesso ao conhecimento em saúde pode ser adquirido por meios formais ou informais e parece ser parte de um processo social e comunitário. A maneira como as informações serão processadas pelos indivíduos dependerá, dentre outras capacidades individuais, do seu nível educacional. Cabe aos serviços e políticas de saúde prover informações compreensíveis e confiáveis, chegando aos indivíduos de forma que possam aplicá-las adequadamente, diminuindo assim os fatores que estão envolvidos na desigualdade na utilização preventiva de serviços de saúde bucal.

Um estudo de revisão sistemática e metanálise mostrou que moradores da zona rural utilizaram menos os serviços odontológicos regulares quando comparados aos de zona urbana (*odds ratio* = 0,87; IC95% 0,76–0,97)^10^. Além disso, crianças da zona rural podem ter maior chance de faltar a consultas preventivas, quando comparadas com as da zona urbana^9^. No município de Rio Grande, RS, a zona rural possui cobertura total da Estratégia Saúde da Família, caracterizada por prover cuidados primários em saúde à população cadastrada nas unidades de saúde. No entanto, não possui equipes de saúde bucal nas suas unidades básicas, sendo o atendimento feito por unidades móveis de forma esporádica. A presença da equipe de saúde bucal atuando junto com as equipes de saúde da família tem sido apontada na literatura como um fator importante para maior uso de serviços de saúde bucal por crianças de até três anos quando comparadas com aquelas que consultaram as unidades de saúde tradicionais^3^, mostrando a importância da organização dos serviços de saúde respeitando os preceitos da atenção básica.

No presente estudo aproximadamente um terço das crianças teve alguma experiência de cárie. A presença de cárie dentária é o principal motivo para buscar o serviço odontológico^14,28^. Entretanto, a prevenção da cárie depende de uma identificação precoce das crianças em risco e de cuidados preventivos como aplicação de selantes e flúor^29^. Neste estudo, a cárie precoce não esteve associada com o uso regular de serviços odontológicos pelas crianças, confirmando achados de estudos anteriores em que as crianças que utilizam regularmente esses serviços não tiveram suas consultas de rotina ou preventivas motivadas por problemas bucais^5^. Nesse sentido, a média de dentes afetados por cáries em crianças que consultaram por check-up pode ser significativamente mais baixa do que aquelas que consultam por razões específicas. Achados recentes apontam que, conforme aumenta o número de consultas odontológicas e ações educativas de prevenção, há uma diminuição do número de lesões cariosas em crianças, observada tanto em ambientes urbanos quanto rurais^30^.

Todos os estudos possuem limitações e pontos positivos. Dois aspectos positivos do presente estudo devem ser considerados. O primeiro é o fato de ser de base populacional, diferente dos estudos anteriores que avaliaram crianças matriculadas em escolas ou durante as campanhas de vacinação. Foi possível, desse modo, avaliar a diferença entre crianças que estavam na escola e crianças sem acesso a ela. Outro ponto positivo refere-se à coleta dos dados do estudo. Os entrevistadores foram treinados para a aplicação do questionário e treinados e calibrados para a obtenção das informações de saúde bucal, minimizando a possibilidade de viés de informação. Em relação às limitações, as perguntas que avaliaram as exposições principais do estudo podem ter sido limitadas em captar informações de educação em saúde bucal das mães e de que forma estas orientações afetam outros comportamentos em saúde bucal. Outra limitação do estudo é que as informações relacionadas à criança foram relatadas pela mãe, existindo a possibilidade da ocorrência de viés recordatório.

Concluiu-se que pouco mais de 11% dos pré-escolares rurais usaram os serviços odontológicos regularmente. As crianças que frequentam creche ou escola e cujas mães receberam informações prévias sobre o cuidado com a saúde bucal apresentaram maior frequência do uso regular de serviços odontológicos. Esses resultados reforçam a necessidade de se estruturar os serviços de saúde e agir intersetorialmente para garantir o acesso efetivo à saúde bucal por meio das equipes de saúde da família. Além de instruir as mães quanto à importância dos cuidados precoces com a saúde bucal das crianças, também é necessário priorizar as crianças que estão fora do ambiente escolar e dependem essencialmente do cuidado materno.
